# Neuropsychiatric symptoms and associated structural neuroimaging correlates in Mild Cognitive Impairment

**DOI:** 10.1002/alz.095258

**Published:** 2025-01-09

**Authors:** Cheryl Cheuk Yan Y Leung

**Affiliations:** ^1^ Institute of Psychiatry, Psychology & Neuroscience, King’s College London, London, London United Kingdom

## Abstract

**Background:**

Neuropsychiatric symptoms (NPS) are behavioural and psychological manifestations frequently present in dementia and mild cognitive impairment (MCI). NPS are known to be associated with adverse health, cognitive and functional outcomes and increasing needs for hospitalisation and institutionalisation. It is understood that MCI may be an intermediate stage between healthy and dementia states. Therefore, NPS‐related brain alterations found at this prodromal phase may provide insights into the pathophysiology of the symptoms and act as potential biomarkers for future prognosis.

**Method:**

By conducting a single‐centre cross‐sectional study of thirty MCI patients from South London and Maudsley NHS services, participants have undergone demographic and clinical interviews, cognitive and neuropsychiatric assessments using the Clinical Dementia Rating, Standardised Mini‐Mental State Examination, and the Neuropsychiatric Inventory ‐ Clinician Rating Scale, as well as 3 Tesla MRI scans. Cognitive, clinical and demographic data for MCI participants with and without specific NPS symptoms have been compared and using a whole brain analysis approach neuroanatomical regions showing correlations between grey matter (GM) volume and NPS symptom severity scores have been identified.

**Result:**

There is some evidence that higher delusion and hallucination severity scores correlate with reduced GM volume in crus I/II of the cerebellum bilaterally, potentially indicating Dementia with Lewy Bodies (DLB) pathology. Furthermore, it has been found that higher aggression severity scores are significantly correlated with greater GM volumes in the calcarine and pallidum regions, and higher irritability severity scores are significantly correlated with greater GM volumes in bilateral anterior cingulate cortex (ACC) and right middle frontal gyrus. An unexpected finding is that the majority of the GM regions of interest (ROIs) appear to have positive correlation with NPS severity scores.

**Conclusion:**

Structural magnetic resonance imaging (MRI)‐based neuroanatomical correlates for a variety of NPS symptoms were found, even as early as the MCI stage of dementia. The unexpected positive correlation between NPS severity and GM for many NPI‐C domains points to possible GM hypertrophy or relative GM sparing associated with NPS. Such volumetric changes may form a distinct recognisable pattern, with potential to increase the specificity of structural MRI biomarkers in predicting future clinical outcomes in MCI.

